# Supplementary material for: Modelling Skylarks (Alauda arvensis) to Predict Impacts of Changes in Land Management and Policy: Development and Testing of an Agent-Based Model
Source: PLoS One. 2013 Jun 6;8(6):e65803. doi: 10.1371/journal.pone.0065803 (PMC3675089; doi:10.1371/journal.pone.0065803)
Supplement: Supporting Information S4 — The skylark ODdox as a zipped archive. (ZIP) [file pone.0065803.s004.zip › Skylark_ODdox/class_position_map-members.html]

ALMaSS Skylark ODdox: Member List


ALMaSS Skylark ODdox
 2.0


- Main Page
- Related Pages
- Classes
- Files

- Class List
- Class Index
- Class Hierarchy
- Class Members

PositionMap Member List

This is the complete list of members for PositionMap, including all inherited members.

|  |  |  |
| --- | --- | --- |
| BitCount(PointerInt n) | PositionMap | inlineprotected |
| ClearMapValue(unsigned x, unsigned y) | PositionMap |  |
| GetMapDensity(unsigned x, unsigned y) | PositionMap |  |
| GetMapDensity(unsigned x, unsigned y, unsigned range) | PositionMap |  |
| GetMapDensity32(unsigned x, unsigned y) | PositionMap |  |
| GetMapDensity5x5(unsigned x, unsigned y) | PositionMap |  |
| GetMapPositive(unsigned x, unsigned y, unsigned range) | PositionMap |  |
| GetMapPositiveB(unsigned x, unsigned y, unsigned range) | PositionMap |  |
| GetMapValue(unsigned x, unsigned y) | PositionMap |  |
| GetTotalN(int x, int y) | PositionMap |  |
| GetTotalSpace(int x, int y) | PositionMap |  |
| Init() | PositionMap | protected |
| m\_ALandscape | PositionMap | protected |
| m\_maxx | PositionMap |  |
| m\_maxy | PositionMap |  |
| m\_TheBitMaskArray | PositionMap |  |
| m\_TheBitMaskArray2 | PositionMap |  |
| m\_TheMap | PositionMap |  |
| m\_xmaxx | PositionMap |  |
| PositionMap(unsigned a\_size) | PositionMap |  |
| PositionMap(Landscape \*L) | PositionMap |  |
| SetMapValue(unsigned x, unsigned y) | PositionMap |  |
| ~PositionMap() | PositionMap |  |


- Generated on Thu Jan 10 2013 13:15:37 for ALMaSS Skylark ODdox by
   1.8.1.1
